# A rare case of lung metastasis from a malignant adenomyoepithelioma of the breast: histological features and therapeutic implications

**DOI:** 10.3332/ecancer.2013.372

**Published:** 2013-11-12

**Authors:** Fausto Maffini, Giuseppe Renne, Rocco Olivadese, Piergiorgio Solli, Marzia Locatelli, Giancarlo Pruneri, Massimo Barberis, Giuseppe Viale

**Affiliations:** 1 Division of Pathology, European Institute of Oncology, Milan 20141, Italy; 2 Division of Thoracic Surgery, European Institute of Oncology, Milan 20141, Italy; 3 Division of Medical Oncology, European Institute of Oncology, Milan 20141, Italy; 4 University of Milan School of Medicine, Milan 20141, Italy

**Keywords:** malignant adenomyoepithelioma, breast carcinoma, lung metastasis

## Abstract

We present a rare case of lung metastasis from a malignant adenomyoepithelioma of the breast. A 44-year-old woman was surgically treated in 2008 for a malignant adenomyoepithelioma of the breast. Shortly after, two lung nodules were detected through a CT scan, and a diagnosis of malignant adenomyoepithelioma was rendered.

## Introduction

The malignant adenomyoepithelioma is a rare tumour of the salivary type in the breast, reported in the literature as a low-grade malignant neoplasm. Distant metastases to the lungs, brain, and liver have been described [1–4].

In this case report, we present a patient with a lung metastasis from a malignant adenomyoepithelioma of the breast one year after the surgical excision of the primary tumour. We describe the histological features of this tumour, and we focus our attention on its therapeutic implications.

## Case presentation

A 44-year-old woman was surgically treated in 2008 for a malignant adenomyoepithelioma of the breast. The pathological staging was pT1c, pN0, M0; the receptor status was oestrogen receptor (ER) lower than 10%, progesterone receptor (PgR), Her-2/neu negative; the proliferative index (Ki-67) was 20%. She underwent a chemotherapy scheme with fluorouracil, epirubicin, and cyclophosphamide (FEC) for six cycles, and, after that, radiotherapy (RT) was performed on the breast neoplastic bed. At follow-up, one year later, a lung nodule suspicious for malignancy was detected via CT scan. In 2009, she again underwent an open lung biopsy with a diagnosis of carcinoma with basaloid-adenoidocystic features, suspicious for breast metastasis, followed by chemotherapy with vincristine and fluorouracil (ViFup) scheme. Another peripheral lung nodule was observed in a CT scan in 2013. She was accepted by our institute for a second opinion and underwent a wedge lung resection with a diagnosis of malignant adenomyoepithelioma. The slides concerning the previous breast tumour were reviewed, and a diagnosis of malignant adenomyoepithelioma was rendered. The patient is now free of disease, and no further therapy was given.

## Discussion

**Figure 1.**
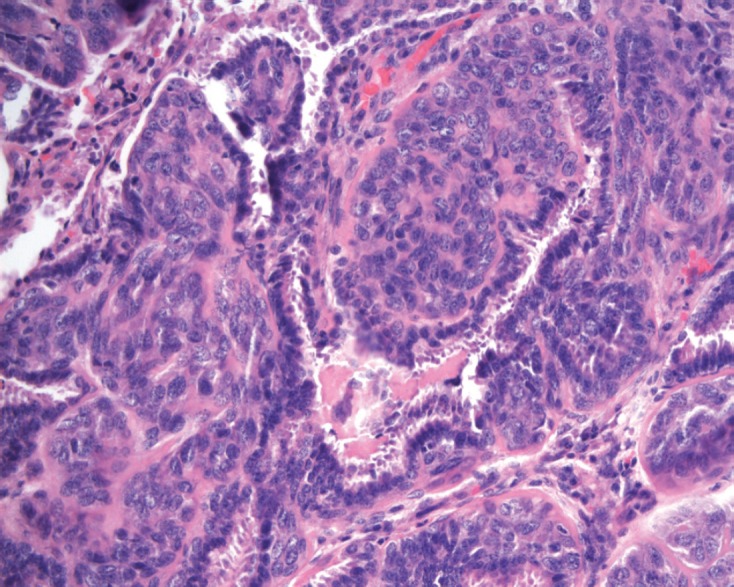
Histological feature of the epithelial-myoepithelial carcinoma, composed by epithelial cells encircled by myoepithelial cells (H&E 20X).

**Figure 2.**
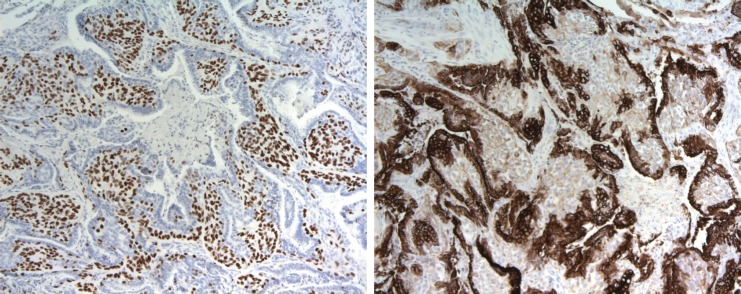
a. P63 nuclear expression in myoepithelial component (p63 10X). b. Cytokeratin 7 expressed by epithelial cells (CK7 10X).

Malignant adenomyoepithelioma is a rare neoplasm of the salivary type in the breast, usually characterised by low malignant potential. Histologically, overtly malignant tumours are characterised by the occurrence of high mitotic activity [more than three mitoses in 10 high power field (HPF)], necrosis, severe cytological atypia, and an infiltrative pattern of growth [4, 5]. The neoplasm is composed of a nest lined externally with myoepithelial cells (Figure 1), expressing S100, calponin, and p63, whereas the internal epithelial cells express cytokeratin 7 (Figure 2a, b). The rare primitive salivary type of lung myoepithelial carcinoma arises in the hilar region and the bronchial wall, as reported. In fact, coin lesions in peripheral lung parenchyma should be considered as having metastatic origins and rarely reported as primitive myoepithelial carcinoma [6, 7]. The relative amount of myoepithelial and epithelial cells is in constant, and tumours composed mainly of epithelial cells may be erroneously diagnosed as infiltrating duct carcinoma, leading to avoidable local and systemic treatments. In fact, although its rarity hindered the establishment of therapeutic guidelines, malignant adenomyoepithelioma is a low malignant potential tumour, whose sensitivity to RT and chemotherapy is questionable [8, 9].

## Conclusion

This case underlines the importance of a correct histological diagnosis of the neoplasm. The patient was subjected to unnecessary therapies, with potentially dangerous side effects. She is now alive and free of disease; a short follow-up is continuing.
